# MRI of total hip arthroplasty: technical aspects and imaging findings

**DOI:** 10.1186/s13244-024-01717-5

**Published:** 2024-06-20

**Authors:** Domenico Albano, Simone Pansa, Carmelo Messina, Salvatore Gitto, Francesca Serpi, Stefano Fusco, Federico Midiri, Luigi Zagra, Luca Maria Sconfienza

**Affiliations:** 1 IRCCS Istituto Ortopedico Galeazzi, Milano, Italy; 2 Dipartimento di Scienze Biomediche, Chirurgiche ed Odontoiatriche, Università degli Studi di Milano, Milano, Italy; 3 Scuola di Specializzazione in Radiodiagnostica, Università degli Studi di Milano, Milano, Italy; 4 Dipartimento di Scienze Biomediche per la Salute, Università Degli Studi di Milano, Milano, Italy

**Keywords:** MARS, Total hip arthroplasty, Aseptic loosening, Infection, Pseudotumor

## Abstract

**Abstract:**

Total hip arthroplasty (THA) is the best surgical approach for treating advanced hip degeneration, providing pain relief, and improved function in most cases. In the past, MR imaging quality has been highly compromised by in-plane distortions, inadequate fat saturation, and other artifacts due to metal components of THA. Technological advancements have made pathologic conditions, which were previously hidden by periprosthetic artifacts, outstanding features due to the optimization of several sequences. To date, several short and long-term complications involving bony and soft-tissue structures may be detected through magnetic resonance imaging (MRI). The use of MRI with adapted sequences and protocols may drastically reduce artifacts thereby providing essential pre-operative elements for planning revision surgery of failed THA. This review has the purpose of conveying new insights to musculoskeletal radiologists about the techniques to suppress metal-related artifacts and the hallmark MRI findings of painful THA.

**Critical relevance statement:**

Advancements in metal-suppression have given radiologists the opportunity to play an emerging role in THA management. This article provides technical and imaging insights into challenges that can be encountered in cases of THA, which may present complications and characteristic imaging findings.

**Key Points:**

- Imaging total hip arthroplasty requires adapted MRI protocol and awareness of the common complications.
- We have reported the available metal-suppression sequences for evaluating total hip arthroplasty.
- Many structures and conditions should be considered when dealing with painful aseptic or septic arthroplasty.

**Graphical Abstract:**

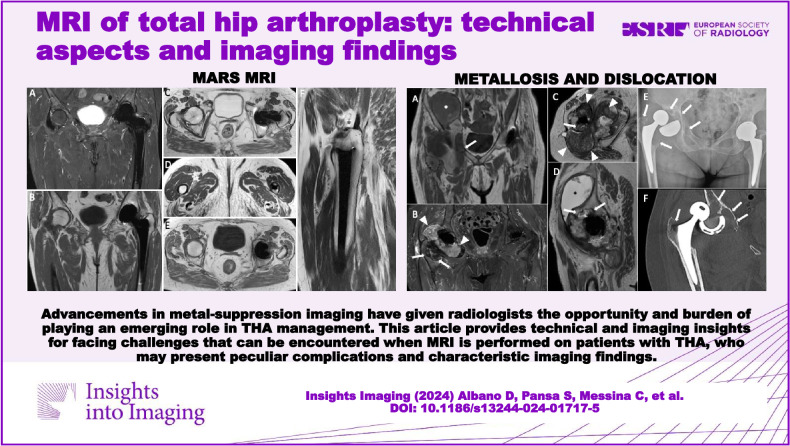

## Introduction

The conventional surgical approach for advanced hip joint degeneration is total hip arthroplasty (THA), which provides pain relief and functional improvement in most cases. The frequency of THA has been rising year after year as a result of the increasing longevity of implants and patients’ longer life expectancies. Despite increased longevity, implants still fail in up to 40% of cases over time [1]. Accurately identifying symptoms linked to THA implants and developing a method to keep track of patients who are at risk are in high demand. The differential diagnosis of painful THA includes several conditions. Unfortunately, the final diagnosis can be challenging, requiring a combination of clinical features, imaging examinations, blood tests, joint aspiration and cultures, and histological examination of intraoperative samples [2, 3]. Pain is the most common symptom in both infected and non-infected failed THA, and typical clinical findings of infected THA are barely observed [4, 5].

Conventional radiography and computed tomography (CT) routinely assist clinical assessment of painful THA for a comprehensive evaluation of possible causes of pain, including periprosthetic fracture, dislocation, breakage of THA elements, loosening, and prosthetic joint infection (PJI), with the latter showing periostitis as a highly specific (about 100%) but scarcely sensitive (about 15%) imaging finding [6]. Also, CT is almost invariably done before revision surgery to evaluate bone stock and to plan prosthetic replacement of the joint. Bone scans are less used than in the past, but they still maintain their space in the diagnostic work-up of painful THA. In this setting, several recent studies have investigated the diagnostic performance of magnetic resonance imaging (MRI) in failed THA [7–12]. Increasing evidence supports the use of MRI for evaluating periprosthetic bone and soft-tissue changes related to painful THA. While in the past, susceptibility artifacts related to the prosthesis itself limited the application of this imaging modality, relatively novel MRI sequences, high-performing scanners, and powerful coils allow for remarkable suppression of metal-related artifacts, thereby providing excellent diagnostic images [13].

This review has the purpose of conveying new insights to musculoskeletal radiologists about the techniques to suppress metal-related artifacts and the hallmark MRI findings of painful THA.

## Artifacts and metal-suppression sequences

**Fig. 1:**
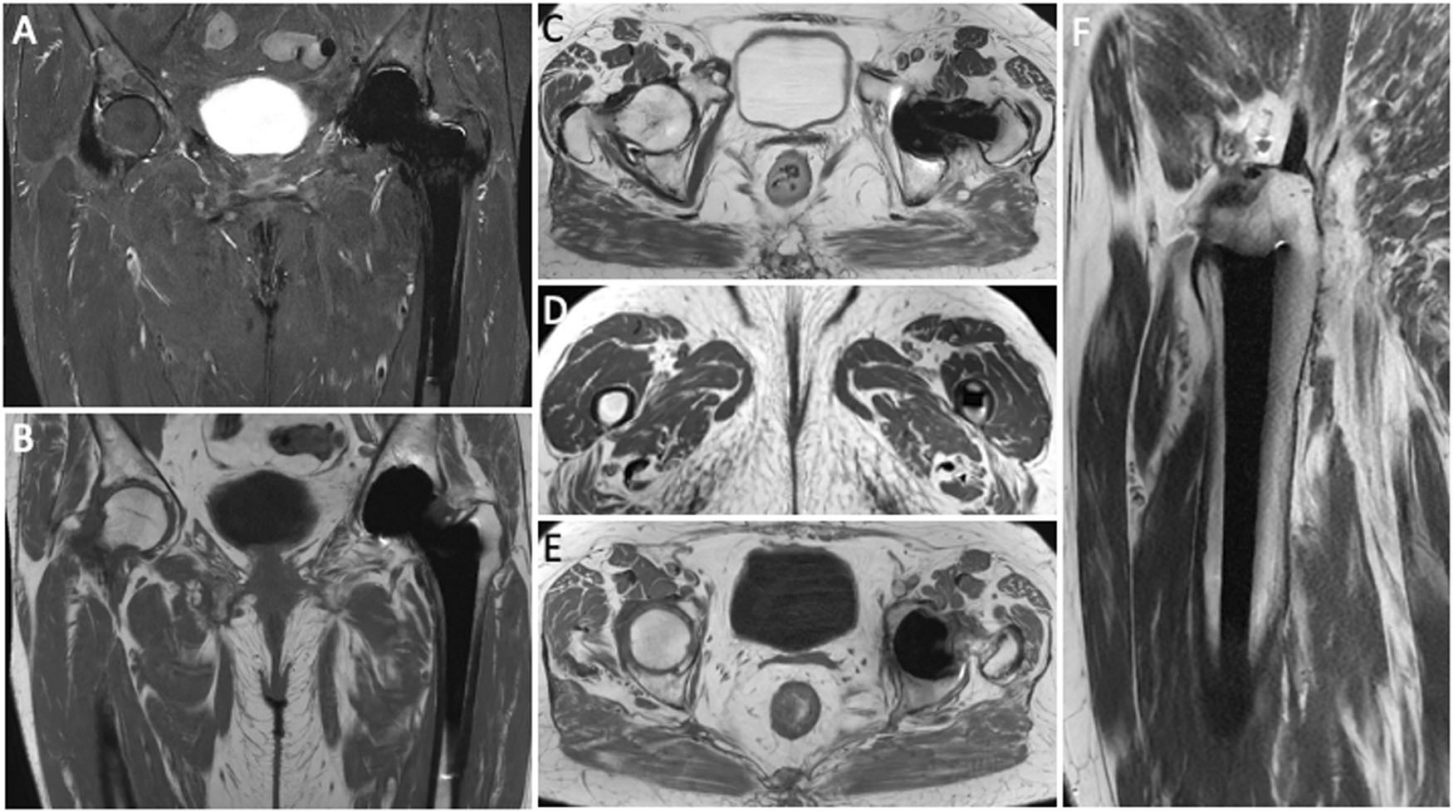
Pelvis MARS MRI of an asymptomatic 69-year-old female with normal left THA. Coronal STIR (**A**), coronal T1-weighted (**B**), axial T2-weighted (**C** and **D**), axial T1-weighted (**E**) and sagittal T2-weighted (**F**) images. No distortion or incomplete fat suppression is observed in these images. Periprosthetic bone is well-depicted without edema or osteolysis. MR images do not highlight other signs of painful THA like effusion, synovitis, collections, pericapsular edema, or masses. Further, no enlarged loco-regional lymph nodes are seen

**Fig. 2:**
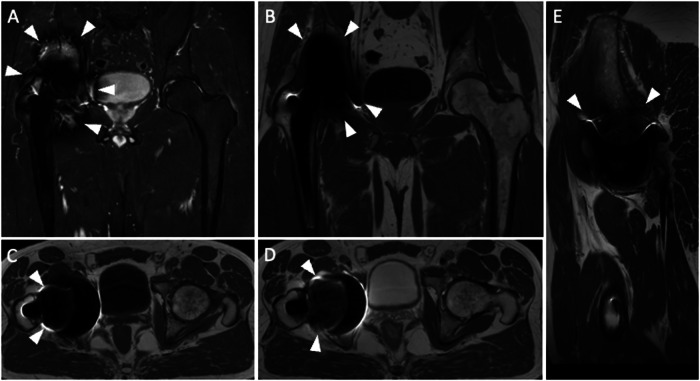
Pelvis MARS MRI of a 71-year-old male with right THA. Coronal STIR (**A**), coronal (**B**), axial (**C**) T1-weighted, axial (**D**), and sagittal (**E**) T2-weighted images are poorly diagnostic due to artifacts and incomplete fat suppression (both indicated by arrowheads) in the periprosthetic areas, particularly around the acetabulum. These artifacts could not be removed even using MARS sequences

The problematic artifacts caused by metal components of THA are in-plane distortions, which result from local field inhomogeneities determined by THA implants. T2* dephasing mediates the cloud of signal loss near metal components. Further, the disruption of local field homogeneity due to implant ferromagnetic characteristics results in a lack of or incomplete fat suppression. Metals in a voxel push the pixels to extend across a wider area, thereby distorting the image. Nevertheless, MRI is now a feasible and accurate modality for imaging THA due to the introduction of new hardware and pulse sequences. Specifically, it is possible to optimize conventional pulse sequences to obtain metal artifact reduction sequences (MARS). Olsen et al [14] introduced the MARS sequence, the first comprehensive method for a metal-suppression sequence. The original sequence design included higher bandwidth, thin section selection (between 3 and 4 mm), longer echo trains, closer echo spacing, and a larger image matrix (Fig. 1). Furthermore, increased gradient strength inversely affects susceptibility artifacts, leading to its reduction. Indeed, employing a broader receiver bandwidth and reducing voxel size, thereby minimizing intra-voxel variation and dephasing, enhances the strength of frequency-encoding and slice-select gradients, consequently reducing distortion and enhancing spatial resolution [15]. Then, modifying the orientation of the frequency-encoding gradient to align with the THA axis will further mitigate susceptibility artifacts [16]. Moreover, for THA imaging, it is advisable to utilize the short tau inversion recovery (STIR) sequence for fat suppression. This is because inversion recovery fat suppression is less affected by magnetic field nonuniformity compared to frequency-selective fat suppression techniques. STIR allows obtaining a superior fat suppression than Dixon, but the latter may be used for obtaining fat suppression facilitating useful post-contrast images. Another way to obtain contrast-enhanced MRI in THA patients is by subtracting post-contrast T1-weighted to pre-contrast T1-weighted images. In addition, opting for imaging at 1.5 T instead of 3 T is advised due to the correlation between susceptibility artifacts and the intensity of the magnetic field utilized. However, even the MARS technique may result in substantial artifacts that can strongly affect image quality (Fig. 2). As a matter of fact, THA material, dimensions, morphology, and position (less artifacts when the long axis of THA is parallel to the magnetic field) impact artifact generation on MRI images. Regarding the material composition of prostheses, it is crucial to note that cobalt exhibits ferromagnetic properties, while chrome and molybdenum do not. Additionally, titanium and ceramic implants are non-ferromagnetic. Indeed, cobalt-chrome prostheses have been shown to generate the most significant artifacts on MRI.

**Fig. 3:**
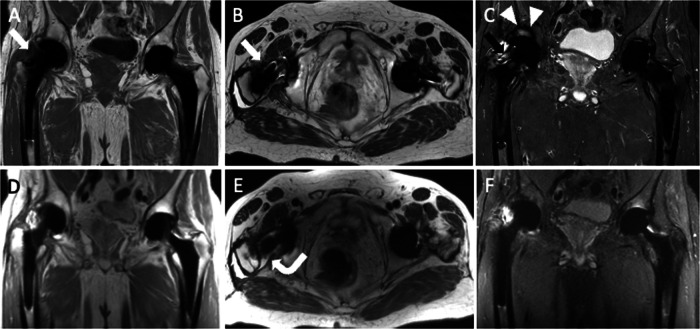
Pelvis MRI performed with MARS (**A** coronal T1-weighted; **B** axial T2-weighted; **C** coronal STIR) and MAVRIC (**D** coronal proton density-weighted; **E** axial T2-weighted; **F** coronal STIR) sequences. MAVRIC images are a bit blurred if compared with MARS, but reduce artifacts and signal loss improving imaging clarity close to metal components. Note the signal loss (arrows) near the prosthetic neck (**A**, **B**, **C**) and poor fat suppression in the acetabulum (arrowheads, **C**) on MARS images. Also, MAVRIC is more effective in depicting the right THA effusion (curved arrow, **E**)

Further dedicated sequences have been developed over time to decrease metal-related artifacts and to better highlight pathological changes of bony and soft tissues that were hidden by artifacts in the past, including WARP (Siemens Healthcare, Munich, Germany), slice encoding for metal artifact correction (SEMAC), and multiacquisition with variable-resonance image combination (MAVRIC). The idea behind the creation of WARP is the implementation of MR imaging provided by MARS, also incorporating to further lessen in-plane distortion. Cho et al [17] first described view angle tilting (VAT), which is now a well-known and widely used method for reducing in-plane artifacts. A further gradient is applied while reading the signal in the slice-select direction. The pixels of interest experience a shearing effect due to this gradient. Hence, there is a section that appears to be slightly tilted. The off-resonance effects are eliminated by the VAT gradient, which equals the excitation. Nevertheless, the main drawback of VAT addition is image blurring. SEMAC is optimal for minimizing through-section distortion. Basically, SEMAC is a 2D fast (or turbo) spin-echo sequence, with all sections having a third-dimensional phase encoding [18]. All the overlapping sections’ third dimensions, or Z-phase encoding, provide a thorough map of the exact ways that magnetic susceptibility has warped the image. These through-section distortions are then corrected, and they are shifted to their correct positions within the final image, using sophisticated reconstruction algorithms. The main limitation of this sequence is the necessary increase in acquisition time. Recently, SEMAC-MRI has been shown to be effective in excluding prosthesis loosening when compared to conventional MRI, showing a sensitivity of 73–91% using T1-weighted SEMAC and STIR SEMAC [18]. Through-section and in-plane artifacts can both be addressed by a second multispectral method, namely MAVRIC (Fig. 3). It is a spin-echo-based sequence based on multiple multidirectional VATs, frequency-selective excitations, computational post-processing, and a conventional three-dimensional readout [19]. The first step is to apply a frequency-selective excitation and refocusing for exploring and mapping a particular frequency range over the field of interest. Several section-encoding steps are often necessary given that the frequency range is generally narrow in comparison to the field of interest being interrogated. Similar to what happens in WARP, multidirectional VAT is employed to decrease the artifacts. As a result, metal-related artifacts dramatically decrease, although two main trade-offs are the substantial increase in image acquisition time and specific absorption rate (SAR). Due to SAR specifications, this sequence can be highly effective at 1 T unit [13]. Hallmarks and limitations of all the above-mentioned sequences are resumed in Table 1.

**Table 1:** Technical hallmarks and limitations of metal-suppression sequences

Sequence	Hallmarks	Limitations
MARS	• Increased section-select • Increased bandwidth • Thin section selection • Longer echo trains closer echo spacing • Larger image matrix	Incomplete in-plane distortion
WARP	• Introduce multidirectional VAT to reduce field inhomogeneities	Blurring of images
SEMAC	• Third-dimensional phase encoding (Z-phase encoding) • Reconstruction algorithms to minimize through-section distortion	Increased time of acquisition
MAVRIC	• Frequency-selective excitations • Multiple multidirectional VATs • Computational post-processing • Conventional three-dimensional readout to decrease in-plane artifacts	Extra-acquisition time and higher SAR

*VAT* view angle tilting, *SAR* specific absorption rate

## THA complications

Periprosthetic fractures, loosening, THA instability, PJI, and unfavorable reactions to prosthetic components are common side effects of THA procedures. Tendinopathies, periarticular soft-tissue ossifications, and neuropathies are additional complications. Hence, a huge number of structures should be evaluated, and several conditions must be considered in patients with painful THA. For assessing periprosthetic bone and detecting bone resorption, CT has been shown to be much more accurate than MARS MRI [20]. Also, dual-energy CT may help reduce metal-related artifacts using two different energy levels and acquiring virtual monoenergetic images that, particularly when combined with the iterative metal artifact reduction technique, ensure significant reduction of image degradation, with virtual monoenergetic images being especially useful for evaluating the metal-bone interface and for decreasing hyperdense artifacts. Nevertheless, CT is less accurate for the evaluation of pseudotumors, collections, and muscle/tendon disorders, although large collections and muscle atrophy/fat infiltration are reliably seen. The effectiveness of MRI and ultrasound for the evaluation of painful THA has been compared with other studies. Ultrasound seems to present lower accuracy for pseudotumor (74%) and muscle atrophy (47–74%) detection [21], although ultrasound may be superior for assessing periprosthetic tendons and effusion.

## Adverse local tissue reaction

One primary factor leading to failed THA is an adverse reaction to prosthetic components, a phenomenon that may lead to pseudotumor occurrence [22]. These reactions may be associated with painful THA and the destruction of periarticular soft tissues, including the capsule, muscles, tendons, and bone [23]. Extensive soft-tissue disruption may determine substantial disability making surgery challenging, and more expensive, with longer rehabilitation needs and worse outcomes [24]. In this regard, an accurate pre-operative planning is crucial. Metallosis and particle disease should be differentiated, given the different pathophysiology and imaging findings, recognized only through MRI.

### Metallosis

**Fig. 4:**
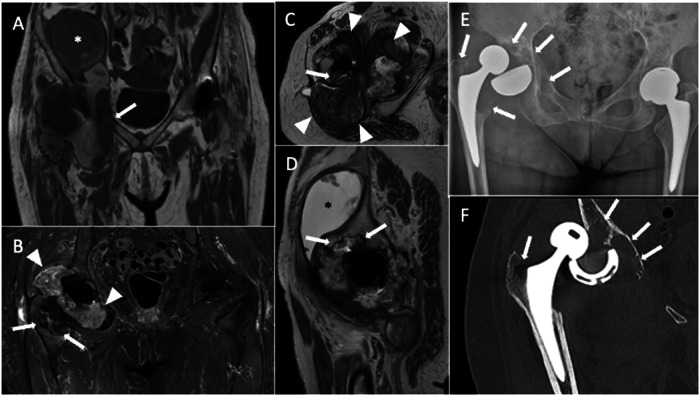
A 90-year-old female with bilateral THA and metallosis on the right. Coronal T1-weighted (**A**), coronal STIR (**B**), axial T2-weighted (**C**), and sagittal T2-weighted (**D**) images show T2-hypointense periprosthetic lobular mass with demarcated low-signal rim (arrowheads) and extensive bone resorption in acetabular, femoral, and superior ileo-pubic ramus (arrows). Also, note the remarkable distension of iliopsoas bursa (*). Five days later, before revision surgery, spontaneous THA dislocation occurred, as shown by plain radiography (**E**) and CT (**F**), which also highlight periprosthetic bone resorption (arrows)

Metallosis is an uncommon state identified by the invasion of metallic debris into periprosthetic soft and bone tissues as a result of THA components degrading. Consequently, patients with metallosis experience periprosthetic bone and soft-tissue necrosis (which is hard to image with MRI), pseudotumor occurrence due to a lymphocytic inflammatory reaction, increased metal ion blood levels, and systemic absorption of wear particles of cobalt (Co) and chromium (Cr) ions that can be detected throughout the body (i.e., brain, thyroid, heart, kidney). Indeed, serum levels of Co and Cr are monitored to screen patients with metal-on-metal THA to identify adverse reactions to metal debris early. The defining feature on MRI is a lobular mass, also called pseudotumor, which exhibits hypointensity on T2-weighted sequences, intermediated-to-high signal intensity on T1-weighted sequences, and a well-defined rim of low-signal intensity on both T1- and T2-weighted sequences [7]. Low-signal foci with blooming due to susceptibility artifacts induced by metal components may be observed in pseudotumors. These masses can be seen everywhere around the THA, particularly in the postero-lateral aspect, inferiorly and anteriorly in the iliopsoas bursa. This slow-growing mass can determine bone destruction and fracture, pelvis extension, compression of neuro-vascular bundles, involvement of subcutaneous tissue up to potential skin ulceration and superinfection (Fig. 4).

### Particle disease

**Fig. 5:**
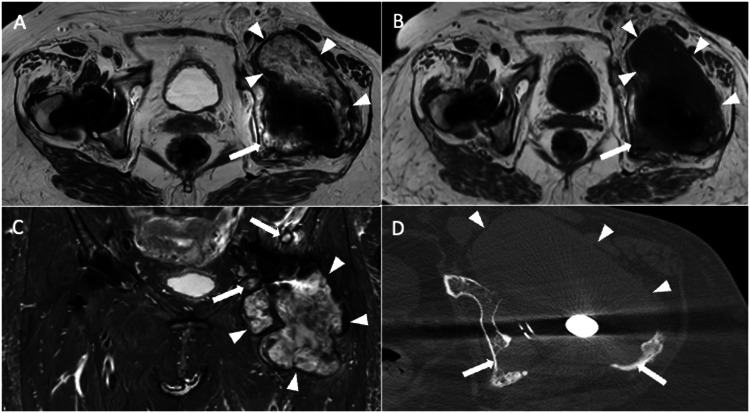
A 73-year-old female with bilateral THA, affected by particle disease on the left THA. Axial T2 weighted (**A**), axial T1 weighted (**B**), Coronal STIR (**C**), and axial bone window CT (**D**) images show high T2/low T1-signal collection with low-intensity rim (arrowheads), also visible on CT image, associated with periprosthetic osteolysis (arrows)

This term includes a group of disorders that stem from the body’s response to microscopic particles of high-molecular-weight polyethylene, cement, or metals, which could be released from THA and enter the nearby tissues. In contrast to metallosis, which results from a typical hapten-mediated type IV hypersensitivity reaction, the particle disease triggers macrophages, potentially leading to inflammatory synovitis and activation of osteoclasts [25]. This process could determine significant bone resorption, fluid accumulations, and painful THA. Then, progressive prosthetic components breaking off induced by this aggressive granulomatous reaction accelerates particle generation further stimulating the progression of disease. Of note, particle wear is expected in THA, but an exaggerated inflammatory response to the wear particles is observed in aseptic loosening. Particle disease presents with effusion and collections showing hyperintensity on T2-weighted sequences, hypointense thickening of the synovium and capsule, and bone resorption. Notably, effusion and synovitis can be found early before the occurrence of symptoms and osteolysis. PJI generally presents T2-hyperintense synovitis without foci or rim of T2-hypointensity. Further, fistulas, pericapsular, and bone edema are typical findings of PJI, not depicted in particle disease. Areas of bone resorption have intermediate signals on T1-weighted imaging, frequently with a hypointense rim around them. Initial features of particle disease include varying synovitis and minor effusions. Osteolysis can then appear as expansile cortical disruption or cortical thinning in particular regions (Fig. 5).

## Periprosthetic fracture

**Fig. 6:**
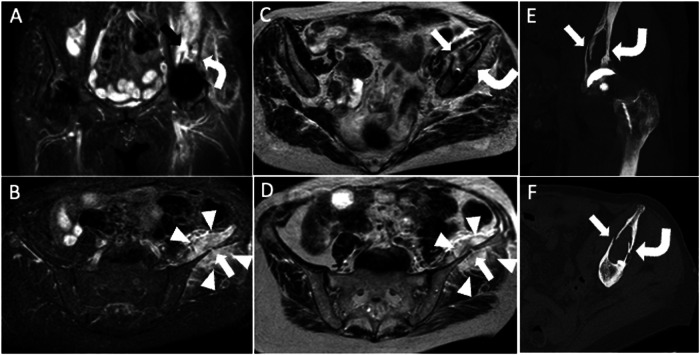
77-year-old female with hip arthroplasty, periprosthetic osteolysis, and fracture. Coronal STIR (**A**), axial STIR (**B**), and axial T2-weighted (**C**, **D**) images show acetabular periprosthetic osteolysis (curved arrows) and fractures that reach the iliac wing (arrows). Also, note the hematoma (arrowheads) within the gluteus minimus and iliac muscles. Coronal (**E**) and axial (**F**) CT images well depict periprosthetic osteolysis (curved arrows) and cortical disruption (arrows)

The risk of death related to periprosthetic fractures is 1.2% for women and 2.1% for men at 70 years of age, while at 80 years is 2.2% for women and 3.9% for men [26]. Periprosthetic fractures can occur during THA placement or post-surgery due to bone resorption, loosening, osteoporosis, stress response, and traumatic events, mostly involving the femur. MRI stands out as the most sensitive approach for measuring the magnitude of periprosthetic bone resorption [27]. MRI of THA can show osseous stress reactions and nondisplaced fracture, comparable to MRI of the native hip. T2-hyperintensity of bone marrow and endosteum, hyperintense thickening of the cortex and periosteum without a clear fracture, and nearby soft-tissue edema are indicators of an osseous stress reaction (Fig. 6). Differential diagnosis of these edema-like changes involving bone marrow, cortex, periosteum, and soft tissues include PJI.

## Osseous integration and aseptic loosening

**Fig. 7:**
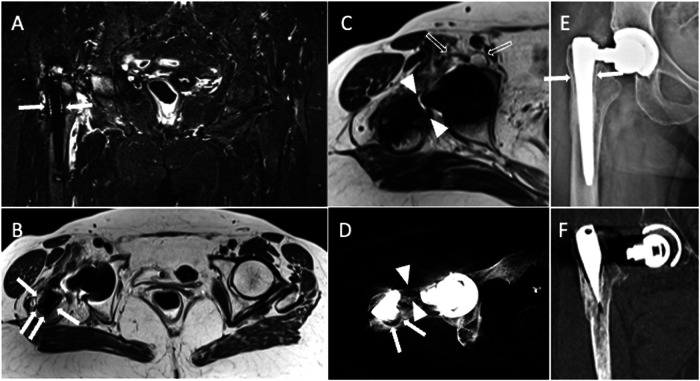
A 77-year-old female with right THA periprosthetic bone resorption and implant rupture. Coronal STIR (**A**), axial T2-weighted (**B**), axial CT (**D**), and AP X-ray view (**E**) images show fibrous membrane and periprosthetic femoral bone resorption (arrows). Axial T2-weighted (**C**) and axial CT (**D**) images show implant neck disruption (arrowheads). Also note ileo-psoas bursitis (void arrows). AP X-ray view (**E**) and coronal CT (**F**) image show supraelevation of the femoral stem

A long-lasting and asymptomatic THA requires optimal fixation into the bone. Despite incomplete integration possibly being acceptable, it is unknown how much bone integration is necessary for THA fixation. Osseous integration will be impeded by the growth of a “fibrous” membrane at the interface between bone and THA components [1, 25]. Osteoclast-stimulating cytokines are released by the synoviocytes through this membrane, which leads to adjacent bone resorption. The complete loss of an implant’s fixation is referred to as mechanical or aseptic implant loosening. When complete integration has been reached, perfect contact of the implant to the surrounding bone is observed. As routinely detected on plain radiography and CT, this fibrous membrane is depicted as bone resorption with a hyperdense soft-tissue layer (thicker than 2 mm) (Fig. 7) [1]. Since the interface between the THA component and bone could be concealed by the implant’s convex surface, which magnifies artifacts, evaluating the integration of acetabular components can be challenging.

## Instability

The primary reasons for revision are THA instability and dislocation (22.5%), loosening (19.7%), and PJI (14.8%) [28]. Component misalignment, THA design, a technique used during surgery, and dysfunction of the abductors are all risk factors for instability. Nevertheless, the extent of soft-tissue dissection during the THA procedure could be the most notable factor. To reduce the risk of posterior dislocation, appropriate reconstruction of tendon insertions after surgery using a posterior approach is needed, since the posterior hip capsule and short external rotator muscle integrity are essential for hip stability. When the posterior capsule is not in contact with the greater trochanter, as well as a gap filled by fluid is observed between the tendons and the greater trochanter, capsular dehiscence and failed reconstruction should be considered [29]. Short external rotator muscle and tendon dysfunction may manifest as muscle atrophy. Because of anterior instability, the anterior hip capsule could experience changes becoming hyperintense and thicker [1]. Component fractures (Fig. 7), acetabular liner displacement, and unsuspected persistent joint dislocation are additional findings following dislocation events.

## Heterotopic ossification

**Fig. 8:**
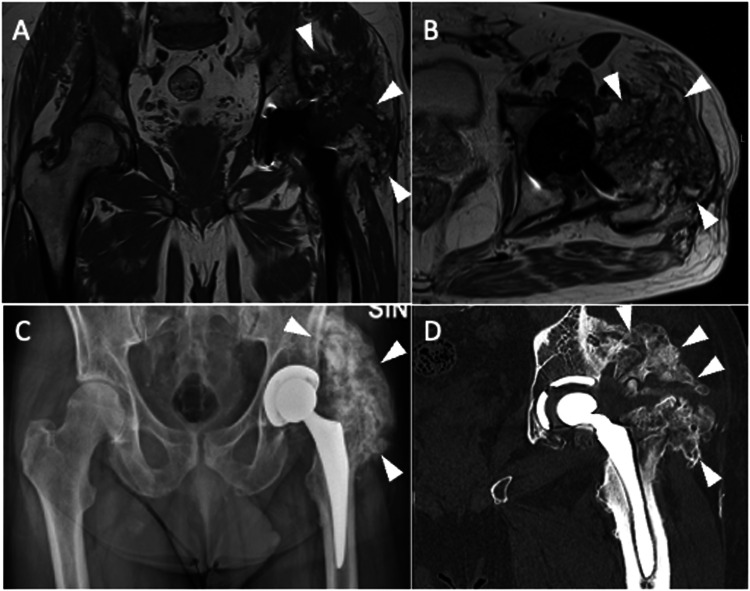
Heterotopic ossification of let THA in a 72-year-old male patient. Coronal T1 (**A**), axial T2-weighted (**B**), AP X-ray view (**C**), and coronal CT (**D**) images show periprosthetic heterogeneous mass with an ossified component which appears hyperintense on both T1- and T2-weighted images (arrowheads). Further, bone resorption is observed along the femoral stem in CT image (**D**)

Heterotopic ossification (HO) refers to the development of bone in tissues that do not typically exhibit ossification-related traits. After THA, 43%–73% [30] of patients experience HO, which is characterized by the development of lamellar bone within nearby soft tissues [31]. Eight weeks following surgery, mature heterotopic bone forms. However, the precise process is unknown. Discomfort, edema, and heating may emerge during the immature osteoid matrix maturation phase, making the clinical picture hard to differentiate from PJI. Immature HO appears as a heterogeneous and irregular expansile pseudomass. To avoid mistaking this appearance for bleeding, tumors, or infectious processes, a heightened level of suspicion is required. HO can be hardly detected on plain radiography in the early phase, but ultrasound is sensitive and can be able to identify suggestive areas of mineralization to reach the correct diagnosis. Mature HO exhibits characteristics of cancellous bone during MRI, including cortex and distinctive bone marrow (Fig. 8).

## Nerve complications

Nerve injury is a rare condition (1%–2%) that should be included among THA complications. The potential mechanisms of injury are nerve stretching, direct injury during surgery or related to THA dislocations, ischemia during implantation, and compression by hematoma or pseudotumors. The sciatic, femoral, and lateral femoral cutaneous nerves are more commonly affected, less frequently compromised nerves are the superior gluteal and obturator nerves. Awareness of surgery technique and access (i.e., anterior for lateral femoral cutaneous nerve injury) is helpful for image interpretation. Typical findings of nerve injury are thickening, signal hyperintensity on fluid-sensitive sequences, the disappearance of neighboring fatty planes, and complete disruption [32]. MRI makes it possible to identify the causes of nerve compression/entrapment or scar tethering of the nerve. Further, muscles may present signs of denervation showing diffuse T2-hyperintensity. MR neurography has proven to be helpful in post-operative settings to detect sciatic nerve lesions in patients subjected to surgery with a posterior approach [33]. However, in several cases, MRI is not able to detect subtle changes in small peripheral nerves, with ultrasound being superior in some settings [34–36].

## Neoplasm

**Fig. 9:**
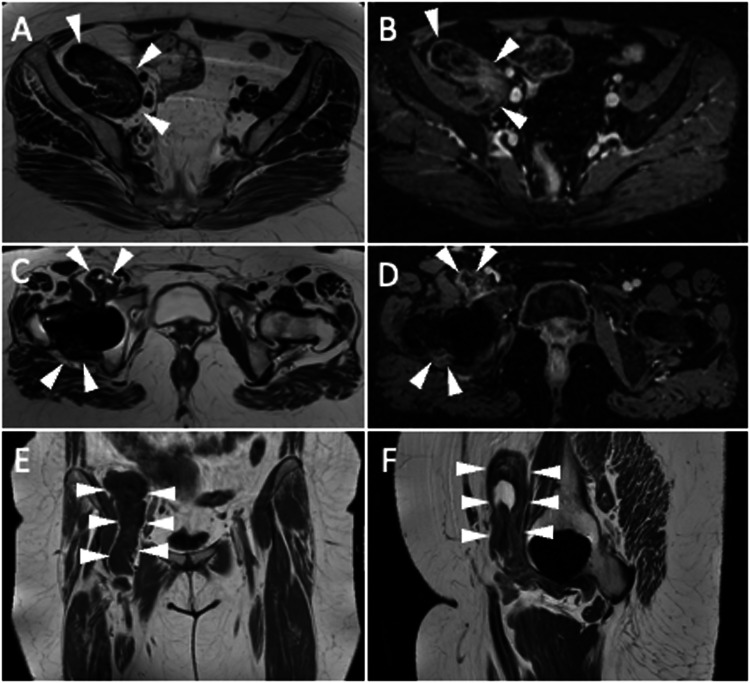
Pelvis MRI of a 58-year-old female who underwent prosthetic replacement of the right hip due to intra-articular tenosynovial giant cell tumor and experienced relapse of disease. Unenhanced axial T2-weighted (**A**, **C**), post-contrast fat-suppressed 3D GRE T1-weighted (**B**, **D**), unenhanced coronal T1-weighted (**E**), and unenhanced sagittal T2-weighted (**F**) images show periprosthetic tissue (arrowheads), located around THA neck and within the iliopsoas bursa, with T1- and T2-hypointensity and strong enhancement, similar to pseudotumor

Although it is uncommon, primary or secondary bone and soft-tissue tumors must be distinguished from other causes of periprosthetic bone resorption and masses. A malignant bone tumor, as in native joints, could appear as a mass growing from the bone and extending into periarticular soft tissues. Soft-tissue tumors can appear as masses with wavy borders that have invaded the nearby bone. A review of 46 malignancies growing from the site of THA showed that sarcomas are prevalent, followed by lymphomas and epidermoid carcinoma [37]. In our experience, metastases are among the most common neoplastic causes of periprosthetic bone resorption. Further, neoplasms that are detected soon after THA are often due to malignancy being overlooked at the time of surgery. A primary tumor of the synovium that can be encountered in THA imaging is the tenosynovial giant cell tumor that can be diffuse or nodular, with low T2 signal intensity, and exhibits extracapsular extension [38]. MRI follow-up after THA for pre-existing tenosynovial giant cell tumor is the optimal imaging tool to find local recurrence and track the development of residual tissue, while tenosynovial giant cell tumor following THA is extremely rare. Of note, contrast-enhanced MRI is generally not used for THA imaging, but it can be helpful in some specific settings like tumors (Fig. 9).

## Muscular and tendon injury

Muscular lesions can be identified and quantified precisely using MRI. Muscular injury is often characterized by myotendinous disruption, fluid accumulation, and architectural disruption or distortion. It holds significance in assessing the degree of architectural disruption, the exact location of muscle tear (proximal-to-distal, myotendinous-myofascial-myoaponeurotic), but also the overall extension of the injury [39]. A hematoma or intra/perimuscular blood products can be the result of bleeding. The main point is the possible muscle damage induced by surgery. Different methods are employed, the posterior (through the gluteus maximus, thereby preserving the gluteus medius and minimus), lateral (through the gluteus medius and vastus lateralis fibers), anterior (through the intermuscular space between the sartorius and tensor fascia latae, thereby preserving muscle status), and anterolateral approaches (through the intermuscular space between the gluteus medius and tensor fascia latae). Recently, minimally invasive surgical techniques have emerged for all of these approaches, aiming to reduce incision length and muscle damage, thereby facilitating post-operative rehabilitation. In this setting, MRI can identify muscle damage, which is especially important in patients experiencing symptomatic abductor ruptures after THA.

**Fig. 10:**
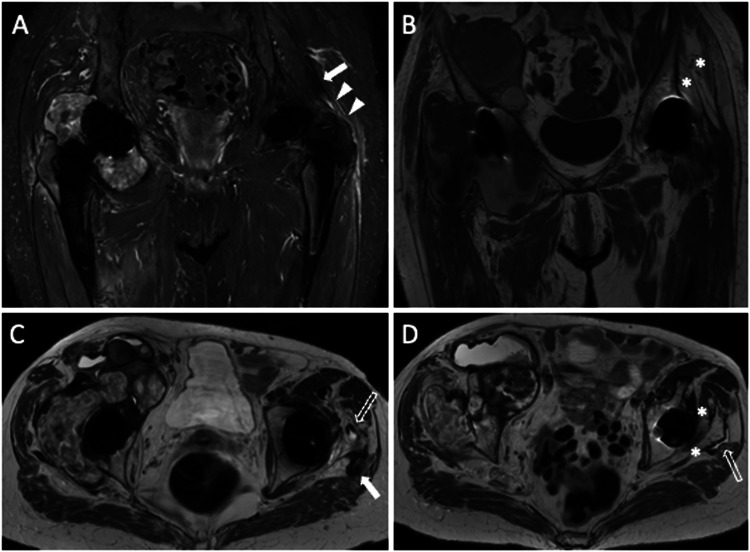
Pelvis MRI of a 69-year-old female patient with bilateral THA and tear of the left gluteus medius tendon. Coronal STIR (**A**), coronal T1-weighted (**B**), and axial T2-weighted (**C**, **D**) images show the complete tear of the gluteus medius tendon (arrowheads: tendon gap; white arrows: tendon stump), atrophy of gluteus medius and minimus muscles (*). Also, peritendinous edema and subgluteal bursitis are observed (void arrows). Note particle disease in the right THA

Groin pain following hip replacement can be determined by tendinopathy and iliopsoas impingement syndrome. Impingement and tendinopathies might be due to a large, protrusive, or misaligned acetabular THA component or acetabular screws [40]. Concomitant iliopsoas and subiliac bursitis may be visible on MRI. Tendinosis, partial-, and full-thickness tears are all on the spectrum of abnormal tendon conditions. Psoas and iliacus muscle atrophy may be a sign of tendon dysfunction or release. The greater trochanteric syndrome could be a source of lateral hip pain presenting with gluteus tendinopathy/tears and bursitis. Clinically, tears in the gluteus medius tendon seem to carry greater significance [41]. Indeed, gluteus minimus is frequently denervated during implantation with its tendon being often released as well, this lessens the clinical significance of minimus tendon tears. MRI shows tendon thickening and hyperintensity in tendinosis. Three types of tendon tears can be described, longitudinal, partial-thickness, and full-thickness tears. Peritendinous soft-tissue edema is frequently seen in these cases (Fig. 10).

## Prosthetic joint infection

**Fig. 11:**
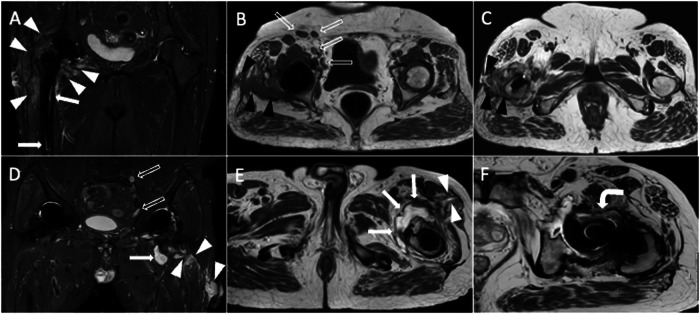
Two cases of infection of the right THA of a 65-year-old female (**A**–**C**) and the left THA of a 73-year-old male (**D**–**F**). Coronal STIR (**A**) image shows pericapsular edema (white arrowheads) and femoral periprosthetic bone edema (arrows). Axial T1-weighted (**B**) and axial T2-weighted (**C**) images show a sinus tract that communicates with the skin (black arrowheads). Increased number and size of loco-regional inguinal lymph nodes (void arrows, **B**) can be observed too compared to the contralateral side. Coronal STIR (**D**) and axial T2-weighted (**E**) images show a periprosthetic fluid collection (arrows) with the sinus tract up to the skin (arrowheads). Also note lamellated synovitis (curved arrow) anterior to the prosthetic neck on axial T2-weighted image (**F**) and enlarged loco-regional iliac lymph nodes on coronal STIR image (void arrows, **D**)

**Fig. 12:**
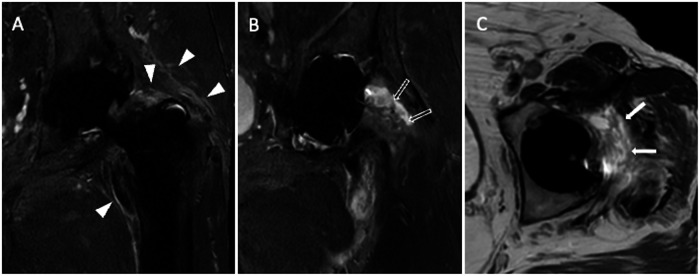
Another case of infection of the left THA of a 69-year-old male. Coronal STIR (**A**, **B**) images show pericapsular edema (white arrowheads, **A**), effusion, and synovitis (void arrows, **B**). Axial T2-weighted (**C**) image shows lamellated synovitis (arrows) close to the prosthetic neck

PJI can show up as vague signs and symptoms, and it may also be associated with pseudotumor conditions, loosening, and soft-tissue damage. The patient can complain of pain only, which is reported in more than two-thirds of patients, but the pain is also frequently observed in patients with non-infected painful THA [5]. On the other hand, more specific clinical findings of PJI like fever and sinus tracts are less common [4]. Laboratory tests can be useful to evaluate inflammatory markers but with limited accuracy in this setting. The standard procedure for diagnosing PJI is joint fluid aspiration with fluid culture. Diagnosis of PJI can be challenging, even using a comprehensive approach including laboratory, clinical, and imaging findings. The detection and identification of periostitis, though regarded as a distinctive characteristic of PJI, is generally made by plain radiography and CT. Frequent Imaging findings of PJI on MRI are pericapsular soft-tissue edema, fluid collections or abscesses, fistulae, effusion and synovitis, bone edema, periostitis, and bone resorptions [42]. Following the injection of gadolinium-based contrast media, diffuse enhancement is observed [13]. Synovial lamellation is strongly suggestive of PJI [43], thickened synovium is generally hyperintense on T2-weighted sequences, differently from T2-hypointensity observed in adverse reactions to metal debris [7]. Notably, it may be difficult to distinguish between intra-articular effusion with debris, thickened and hyperemic synovium, and thickened periarticular soft tissues without intravenous contrast injection. In some cases, post-contrast T1-weighted images can be crucial for the correct interpretation of these findings highlighting the enhancement along the course of the synovium. T2-hyperintense synovitis and lamellated synovitis have been shown to be highly specific (97.5% specificity) MRI findings of infected THA with 90% and 83% positive predictive value but low sensitivity (47% and 26%), respectively [7]. On the other hand, bone edema seems to be the MRI feature with the highest sensitivity (76%) and negative predictive value (88%) for PJI. Bone destruction and osteomyelitis symptoms occur in persistent or aggressive infections. A peculiar imaging finding is the enlargement of loco-regional lymph nodes, that should be compared to the contralateral side to enhance the ability to diagnose PJI (Figs. 11 and 12) [7]. Imaging findings of THA complications are resumed in Table 2.

**Table 2:** Imaging findings of THA complications

Complications	Typical imaging findings
Metallosis	Lobular mass with T2-hypointensity and well-defined hypointense rim, associated with osteolysis
Particle disease	Effusion and collections showing hyperintensity on T2-weighted sequences, hypointense thickening of the synovium and capsule, and bone resorption
Periprosthetic fracture	T2-hyperintensity of bone marrow and endosteum, hyperintense thickening of the cortex and periosteum with fracture line, callus may be observed
Aseptic loosening	Periprosthetic membrane and bone resorption
Heterotopic ossification	Lamellar bone in periprosthetic soft tissue; immature ossification may not present the typical bony appearance
Nerve complications	Thickening of the nerve, loss of perineural fatty planes, internal signal hyperintensity
Muscular/tendon injuries	Muscle atrophy, bursitis, tendinosis with peritendinous edema, partial and full-thickness tears
Infection	Soft-tissue edema, collections, draining sinuses, synovial lamellation, T2w hyperintense synovitis, joint effusion, marrow edema, loco-regional lymphadenopathies

## Conclusions

THA is extremely common and effective, with the average age of patients at implantation getting younger. However, a small fraction of patients require revision surgery due to failed THA. MRI performed with dedicated metal-suppression sequences is an effective modality for identifying the sources of pain following THA implantation. MRI is particularly helpful for evaluating periprosthetic soft tissues in patients with PJI and adverse local tissue reactions to assess and determine the extent of collections, fistulae, and pseudotumors, but also in patients with muscles, tendons, and nerve injuries. Radiologists must be aware of imaging novelties in this field, taking advantage of sequence optimization to produce images of diagnostic quality. To date, the role of radiologists in the identification and management of complications following THA has been growing. Peculiar complications may affect failed THA with characteristic imaging findings that are not observed in native hips. Hence, musculoskeletal radiologists should acknowledge the MRI features of these conditions when dealing with patients with THA.

## Data Availability

The datasets used and analyzed during the current study are available from the corresponding author upon reasonable request.

## Abbreviations

CT: Computed tomography
HO: Heterotopic ossification
MARS: Metal artifact reduction sequence
MAVRIC: Multiacquisition with variable-resonance image combination
MRI: Magnetic resonance imaging
PJI: Prosthetic joint infection
SAR: Specific absorption rate
SEMAC: Slice encoding for metal artifact correction
STIR: Short tau inversion recovery
THA: Total hip arthroplasty
VAT: View angle tilting
